# Force-sensing mobile microrobotic grippers for gentle and precise bioassembly of cell spheroids

**DOI:** 10.1063/5.0304932

**Published:** 2026-04-28

**Authors:** Aaron C. Davis, Madison O'Brien, Iris Gong, Luis Solorio, David J. Cappelleri

**Affiliations:** 1 School of Mechanical Engineering, Purdue University, 205 Gates Rd., West Lafayette, Indiana 47906, USA; 2 Weldon School of Biomedical Engineering, Purdue University, 206 S. Martin Jischke Dr., West Lafayette, Indiana 47906, USA

## Abstract

This paper presents a wireless mobile microrobot gripper for the pick-and-place bioassembly of cell spheroids, which are crucial for tissue engineering. To address the risk of cellular damage during handling, this technology integrates real-time force sensing, enabling controlled manipulation of delicate biological materials. The microscale, untethered design allows for high maneuverability during the construction of complex, multi-spheroid constructs. Experimental results demonstrate the system's effectiveness in creating precise, heterogeneous patterns while maintaining high cell viability. This force-aware microrobotic platform overcomes a barrier in biofabrication, paving the way for the gentle and precise construction of complex tissue models for biomedical engineering.

## I. INTRODUCTION

Three-dimensional (3D) cell cultures, including spheroids (spherical aggregates of cells), are often used for tissue engineering and drug discovery applications because they better mimic *in vivo* cell behavior when compared to two-dimensional (2D) cultures.^1–3^ Spheroids facilitate cell–cell and cell–matrix interactions, allowing them to model the *in vivo* physicochemical environment. Also, 3D cell cultures have greater stability and longer lifespans than 2D cultures, making them more appropriate for long-term drug studies.^4,5^ Spheroids have been used to model oxygen gradients in the cancer microenvironment^6^ and to emulate tissue fibrosis *in vitro*, allowing for advanced analysis of human disease progression.^7,8^ Beyond modeling, cell spheroids have been used for regenerative medicine applications.^9,10^ Although spheroid cultures hold great promise, most cultures are limited to smaller length scales (< 1 mm), and it is difficult to separate and organize heterogeneous cell populations.^11,12^

Larger scale 3D *in vitro* tissue models are often created using biofabrication techniques, which generate structurally organized, biologically functional products.^13^ Commonly used biofabrication techniques, including bioprinting, predominately use cells embedded in hydrogels, resulting in constructs with low cell densities.^11^ Furthermore, polymer scaffolding can cause unwanted matrix signaling and limit important cell–cell interactions.^11,14^ These limitations led to the creation of bioassembly techniques, where preformed cell-containing units are organized and assembled into 3D constructs.^15^ Cell spheroid bioassembly has become of particular interest due to its potential to create complex 3D heterogeneous structures for cell dense tissue models, regenerative medicine, and tissue engineering applications.^16^ When in close proximity with one another, spheroids can fuse to create a larger single body, resulting in a physically larger model.^3^ Recent advances in single-spheroid bioassembly enable precise control over the spatial patterning and resulting construct geometries. These methods include using non-adhesive micromolds to form constructs with unique geometries^9,17^ and the Kenzan method, which skewers spheroids onto metallic needles and allows them to fuse together.^18^ Unfortunately, these methods have a high chance of construct disintegration after removal.^19,20^ Other spheroid bioassembly techniques utilize hydrogels to support fused spheroid constructs,^21,22^ but these methods often mechanically disrupt spheroids or require additional cross-linking that could affect the tissue construct.^20,23^

Several wireless micromanipulation methods look to overcome these issues associated with current bioassembly techniques. Two of the most effective methods for this have been optical manipulation^24,25^ and mobile microrobots.^26–31^ Optical methods, often called optical tweezers, are quite effective in isolating objects and securely manipulating them but are most applicable to single cell manipulation, as the maximum force that can be applied is severely limited ( <1 nN).^32,33^ Microrobotic manipulation methods are much more versatile as they are capable of applying forces >50 *μ*N,^34^ but they generally require more complex fabrication and control systems.

Magnetic actuation of microrobots is particularly appealing for spheroid bioassembly due to the biocompatibility of low-frequency magnetic fields, even at high strengths.^35^ Biological micromanipulation using microrobots has been demonstrated in several different applications, including single cell manipulation using a U-shaped microrobot^36^ and implementing microrobots into a microfluidic device for cell sorting.^37^ However, these methods, which often rely on pushing mechanisms, require highly controlled environments, as they are susceptible to external disturbances. Any unintended flow or movement can disrupt precise manipulation. Multi-robot manipulation methods can overcome this issue by surrounding the object to securely capture it during movement,^38–42^ but require more complex systems to enable parallel control, as standard methods utilize global fields to control microrobots.^26^ Single robot methods of capture are also possible, but generally require multiple actuation methods to enable a gripping or closing action. Fully magnetic actuation for capture is possible, but requires magnetic inhomogeneity within the robot, which is difficult at small scales. Though some work has been done in reducing the size of these devices, reaching close to the 1 mm scale.^43,44^

However, any contact manipulation introduces a significant risk of cell damage or death.^45^ This risk stems from the fundamental principle that living cells are acutely mechanosensitive to their physical environment. External forces—such as compression, shear stress, and tension—profoundly modulate key cellular functions, including proliferation, differentiation, and survival.^46–48^ For proper tissue development, applied forces must be carefully maintained within a narrow physiological window. For example, specific thresholds of compressive stress must be avoided: forces exceeding 5–10 kPa often induce cell death or necrosis,^49^ while even moderate, prolonged stress (1–5 kPa) can paradoxically drive invasive behavior in certain cell types.^50,51^ Moreover, cellular damage is not exclusively caused by large, instantaneous forces; extended exposure to low-magnitude stress or fluid shear can also lead to irreversible cellular damage over time.^52–54^ Therefore, optimizing bioassembly protocols requires not only precise positional control but also an active strategy for managing and minimizing the mechanical forces exerted on the delicate cellular building blocks.

A critical technological gap in microrobotic bioassembly is the pervasive lack of integrated force sensing. Although microrobots have demonstrated positional precision,^36,55^ current open-loop control systems fail to provide real-time feedback on the mechanical interaction forces. This deficiency forces operators to rely on estimates, creating an unacceptable risk of exceeding safe mechanical thresholds and compromising the long-term functional integrity of the engineered tissue. The full potential of high-force microrobotic manipulation can only be achieved by making the process gentle and force-aware.

**FIG. 1.**
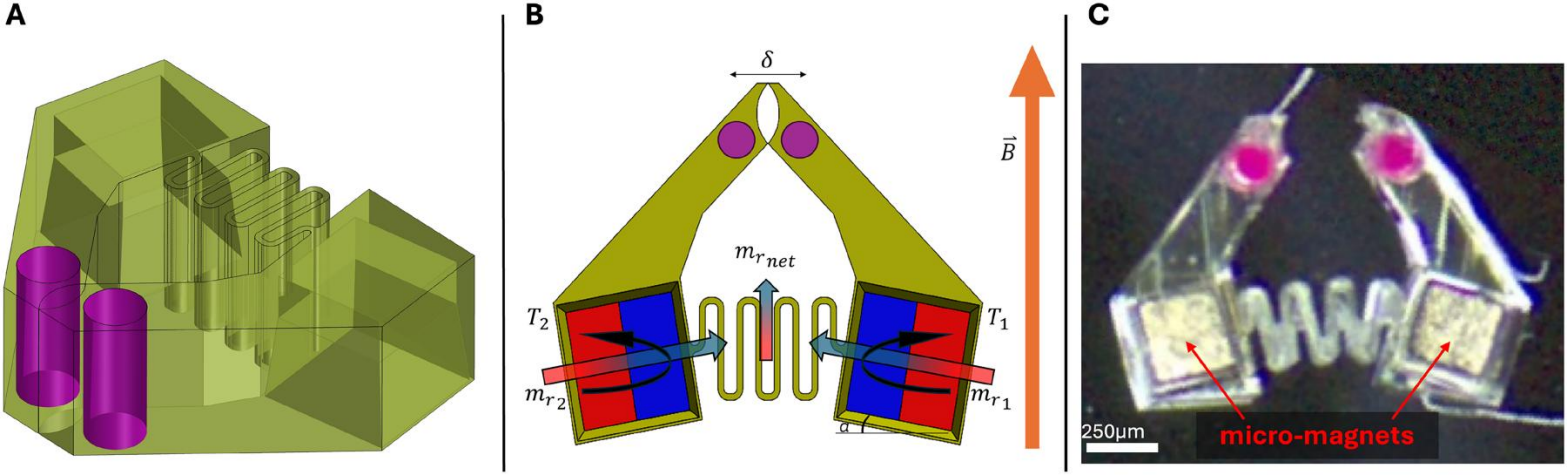
Mobile microrobot gripper (MMG) design and operating principle. (a) The housing of the MMG incorporates receptacles for two 250 μm cubic permanent magnets, a serpentine hinge, and tracking fiducial channels. (b) Free body diagram illustrating the magneto-mechanical interactions in the microrobot. The net magnetic moment $m_{r_{\text{net}}}$ aligns to the applied magnetic field $\vec{B}$, allowing orientation control. The $\vec{B}$ field over a certain threshold generates opposing torques in the two micromagnets causing the serpentine hinge to bend and the gripper jaws to open. Additionally, magnet gradients are used to translate the MMG around the workspace. (c) A microscope image displaying the MMG with the serpentine hinge, micromagnets, and pink colored fiducials visible.

To overcome this limitation, we present a novel wireless mobile microrobot gripper (MMG) system that incorporates real-time gripping force measurement and feedback control (Fig. 1). The MMGs are fabricated using two-photon 3D printing, incorporate a compliant serpentine hinge for controlled and gentle gripping, and are designed with visual fiducials. These fiducials are tracked by a computer vision system to accurately estimate the gripper's opening displacement, which is then correlated with the applied force via mechanical calibration. They are actuated magnetically, allowing for precise manipulation of their orientation, translation, and gripping actions. We found cell viability was not compromised during spheroid manipulation, and a microrobot gripping force of 500 *μ*N did not mechanically deform or damage the cell spheroids. In addition, microrobot-placed spheroids continue to grow and spread after placement on a predefined grid, creating a heterogeneous two-dimensional tissue construct that mimics a 3.06 mm^2^ cell sheet. This approach offers a promising solution for precise and delicate bioassembly tasks, with potential applications in tissue engineering and regenerative medicine.

## II. RESULTS AND DISCUSSION

### A. Magnetic microgripper design and modeling

The underlying principle behind the wireless control of the MMG with embedded magnets lies in the interaction between magnetic fields and magnetic moments. The magnetic force, ${\vec{F}}_{m}$, and torque, ${\vec{T}}_{m}$, acting on a magnet with magnetic moment, $\vec{m_{r}}$, within an applied field, $\vec{B}$, are given by the following equations:

$${\vec{F}}_{m}=({\vec{m}}_{r}\cdot \nabla )\vec{B},$$ (1)

$${\vec{T}}_{m}={\vec{m}}_{r}\times \vec{B}.$$ (2)

These equations demonstrate that both the magnetic force and torque are directly influenced by the magnetic moment of the magnet and the strength and direction of the applied magnetic field.

#### 1. Multi-magnet grippers

In the context of the multi-magnet MMG, these equations are utilized to achieve controlled locomotion, orientation, and gripping actions. The strategic placement of multiple magnets within the MMG allows for the manipulation of its net and internal magnetic moments, creating net and internal forces [Eq. (1)] and torques [Eq. (2)]. Thus enabling precise control over its behavior in response to external magnetic fields.

The MMG is designed to be controlled wirelessly using magnetic fields. The orientation of the MMG is determined by the direction of the net magnetic moment, which is the vector sum of the magnetic moments of the individual magnets within the MMG [Fig. 1(b)]. In the presence of an external magnetic field, the MMG experiences a torque that aligns its net magnetic moment with the field direction [Eq. (2)]. By controlling the orientation of the external magnetic field, the orientation of the MMG can be precisely manipulated. This allows for the alignment of the MMG with the target object. The translation of the MMG is achieved by utilizing magnetic field gradients. The magnetic force acting on the MMG is proportional to the gradient of the external magnetic field [Eq. (1)]. By adjusting the gradient, the MMG can be pulled in the desired direction with forces up to 4.5 *μ*N, enabling the MMG to move toward the target location.

The gripping mechanism of the MMG is actuated by the interaction of the external magnetic field with the individual magnetic moments of the embedded magnets. During fabrication, the two magnets are aligned with a 10° offset from directly opposing each other. This creates a net magnetic moment that is misaligned from the internal magnetic moments. As shown in the free body diagram in Fig. 1(b), the opposing torques experienced by these magnets under an applied magnetic field induce internal stresses within the MMG's structure. The MMG's design incorporates a compliant serpentine hinge that allows significant deflection, enabling the gripping action under a controlled magnetic field. When exposed to the magnetic field, the relative alignment of the embedded permanent magnets generates magnetic torques that cause the opening and closing of the grippers. The magnitude of the applied magnetic field, ranging from 1 to 16 mT, determines the extent of the gripping action.

#### 2. Gripper response

**FIG. 2.**
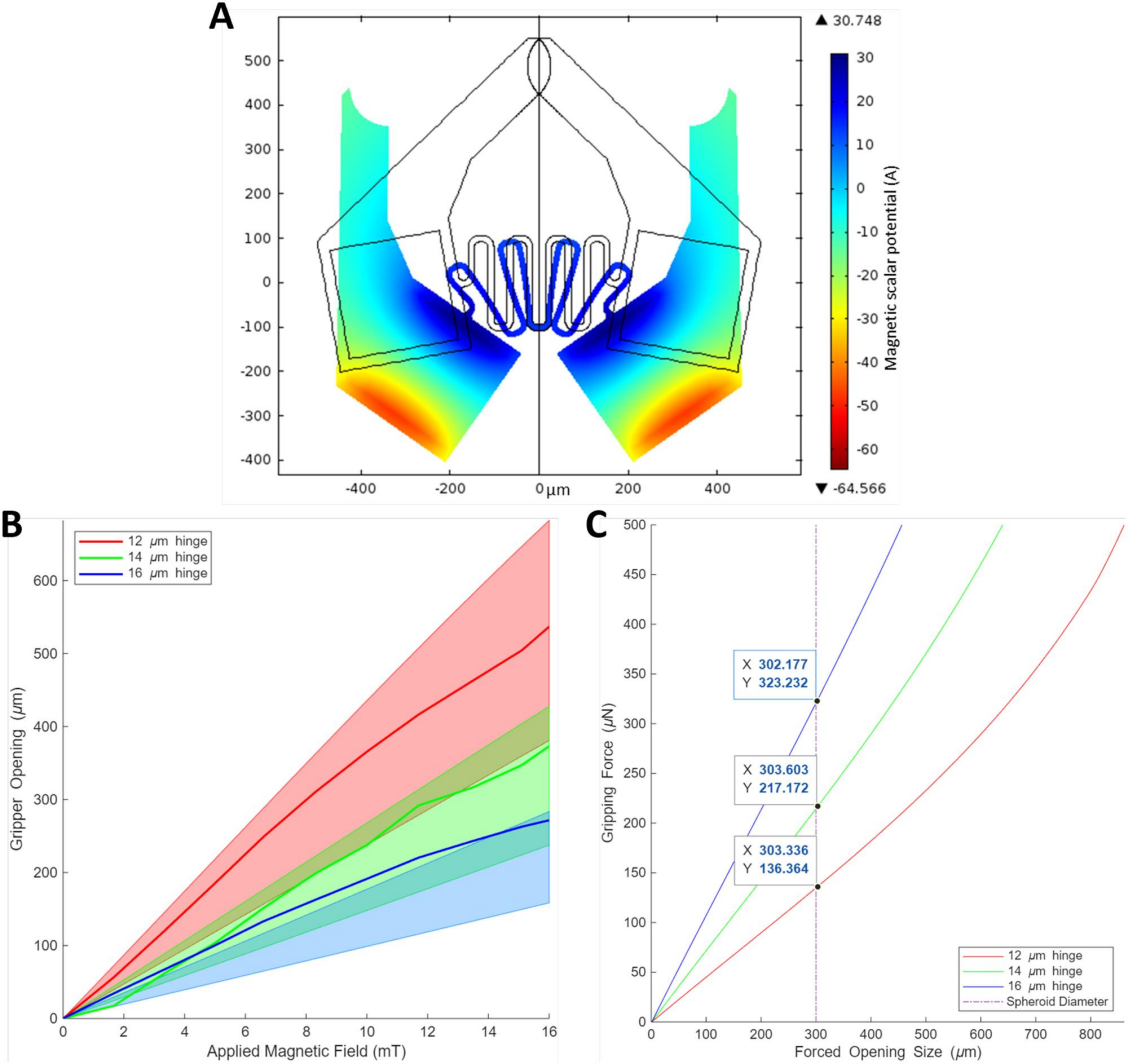
Numerical and experimental results of gripping action. (a) FEA model showing gripper actuation under a 16 mT applied magnetic field with magnetic scalar potential shown in color. (b) Gripping response of MMGs with 12, 14, and 16 *μ*m thick serpentine hinges. Shaded regions indicate the range of openings for grippers with different strength magnets ( 714±196 m) based off of the FEA model. Lines mark the gripper opening experimental data. (c) Gripping force as a function of forced opening size for various serpentine hinge widths (12, 14, and 16 *μ*m). The vertical line indicates a 300 *μ*m diameter spheroid, with corresponding gripping forces of 136, 217, and 323 *μ*N for the respective hinge widths. This analysis highlights the relationship between hinge size, gripping force, and potential applied force on manipulated objects, such as delicate cell spheroids.

A thorough analysis of gripper actuation was performed using a combination of finite element analysis (FEA) and experimental measurements. A detailed FEA model was developed to simulate the gripper's response to the applied magnetic field [Fig. 2(a)]. The model incorporated the material properties of the 3D-printed structure, the magnetic properties of the embedded magnets, and the strength of the external magnetic field. To validate the FEA model, a series of experiments was conducted using fabricated MMGs. The grippers were actuated under varying magnetic field strengths, and the resulting gripper opening displacements were measured using a microscope camera.

To analyze the gripper's response to varying design parameters, we investigated the impact of serpentine hinge width and magnet strength on gripper opening actuation. FEA simulations and experimental measurements were conducted for hinge widths of 12, 14, and 16 *μ*m. A clear correlation was observed between hinge width and gripper opening displacement: as hinge width increased, the displacement decreased for a given magnetic field strength. All three hinge widths exhibited linear behavior within the tested range. However, this linearity deviates at larger opening sizes as the legs of the serpentine hinge come into contact, decreasing the active bending length and effectively increasing the hinge's stiffness [Fig. 2(b)].

The influence of magnet strength on gripper response was also examined. We measured the magnetic strength of the micromagnets to be 714±196 mT and incorporated these data into our FEA model to explore the effect of deviations from the expected magnetic strength. The results aligned with simplified mathematical models, showing a linear relationship between gripper opening size, applied magnetic field strength, and magnet strength. It should also be noted that the printed IP-S resin exhibits viscoelastic behavior under certain loads, which was not modeled. This can lead to increased jaw opening sizes and decreased gripping force as the material relaxes.

To ensure the safety and viability of cell spheroids during manipulation, we analyzed the gripping force using our validated FEA model. This analysis determined the maximum force exerted by the gripper when the gripper was forced open as a function of hinge width and opening size [Fig. 2(c)]. For a 300 *μ*m diameter spheroid, the gripping forces at the corresponding opening size are 136, 217, and 323 *μ*N for the 12, 14, and 16 *μ*m hinges, respectively. This demonstrates that the maximum gripping force can be tuned by adjusting the hinge width. To balance gripping strength and opening size, the 14 *μ*m hinge design was used for the work in Secs. II B–II D.

### B. Real-time gripping force estimation

As described in previous work,^56,57^ the MMG is designed as a compliant mechanism, allowing the gripping force ( $F_{\text{grip}}$) to be calculated based on the deflection of the serpentine hinge, following Hooke's law ( F=kΔx). Since the magnetic field ( $\vec{B}$) is the sole source of actuation, the total measured opening of the gripper jaws ( $\delta_{m}$) is a superposition of the magnet-induced opening and any displacement caused by contact with an external object (e.g., a cell spheroid).

The force estimation methodology leverages the relationship between the applied magnetic field and the gripper's stiffness,

$$F_{\text{grip}}=k(\delta_{m}-\delta_{B}).$$ (3)

Here, $\delta_{B}$ represents the theoretical opening displacement of the gripper jaws if no object were present, derived from the calibrated magnetic opening coefficient ( $C_{om}$) and the applied magnetic field magnitude ( $\vec{B}$): $\delta_{B}=C_{om}B$. The stiffness constant (k) of the hinge is predetermined using finite element analysis (FEA) as described in Sec. II A 2. The actual measured opening, $\delta_{m}$, is extracted in real-time by the computer vision system tracking the colored fiducials. From this, the gripping force is determined instantaneously by calculating the difference between the expected and measured openings, then multiplying by the known hinge stiffness,

$$F_{\text{grip}}=k(\delta_{m}-C_{om}\vec{B})=k(\delta_{f}).$$ (4)

This calculated force is continuously displayed to the user via the control interface, providing a quantitative metric for monitoring interactions. To ensure safe handling of biological material, the system implements a soft safety threshold; when the estimated force exceeds a predefined limit (e.g., 500 *μ*N), a visual alert is triggered, prompting the operator to reduce the applied magnetic field and mitigate potential cell damage.

**FIG. 3.**
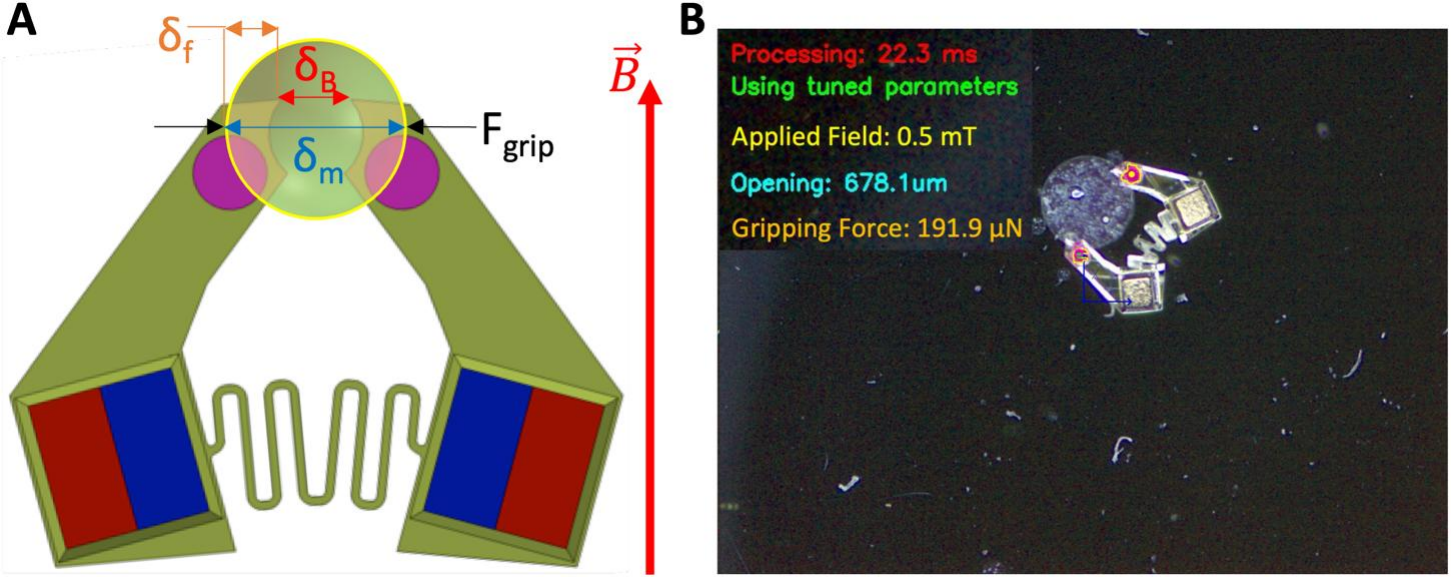
MMG displacement and forces and force–feedback interface. The gripper opening measurement is used in conjunction with the calibrated magnetic response and stiffness curves to calculate the applied gripping force, which is shown in the user interface. (a) For the applied magnetic field $\vec{B}$ the expected gripper opening is $\delta_{B}$. When gripping an object, the visually measured opening is $\delta_{m}$. The difference between the expected and measured opening size ( $\delta_{f}$) is used to calculate the gripping force ( $F_{\text{grip}}$) using Hooke's law [Eq. (4)]. (b) The MMG centroid and the colored fiducials are both tracked in real-time using contour filtering and color masking. This allows accurate, real-time measurement of gripper position, orientation, and opening.

**FIG. 4.**
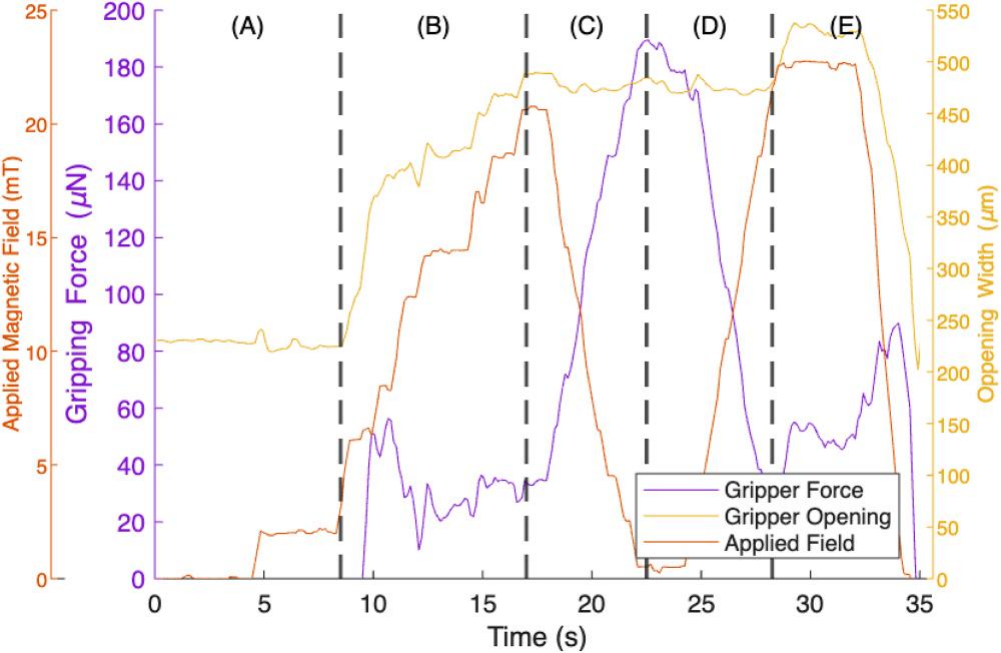
PDMS micro-sphere gripping force measurement. The applied magnetic field ( $\vec{B}$), measured gripper opening ( $\delta_{m}$), and calculated gripping force ( $F_{\text{grip}}$), as related by Eq. (4), when a 500 *μ*m PDMS sphere is grasped by the MMG. (a) t = 0–8 s: a low strength (2 mT) constant magnetic field is applied to the MMG to enable stable control. (b) t = 8–17 s: increasing the strength of the applied magnetic field to 20 mT opens the gripper enough to grab the sphere. (c) t = 17–22 s: the gripper closes on and captures the sphere when the magnetic field is reduced. As the magnetic field decreases, a significant gripping force is applied to the sphere and measured by the vision system. (d) t = 22–28 s: the sphere is released by once again increasing the applied magnetic field. (e) t = 28–35 s: the gripper moves away from the sphere and closes when the applied field is removed.

To test the system, we performed experiments with a 500 μM diameter PDMS sphere, as it is similar in size, shape, and stiffness to the cell spheroids for our target bioassembly application. Figure 3 shows a screenshot of the user interface and real-time force information being displayed to the user during the experiment. Figure 4 shows the real-time measurement data of the MMG as it picks up the 500 μm sphere. It shows the relationship between the applied magnetic field, gripper opening, and gripping force at various points during the experiment.

### C. Cellular system compatibility

**FIG. 5.**
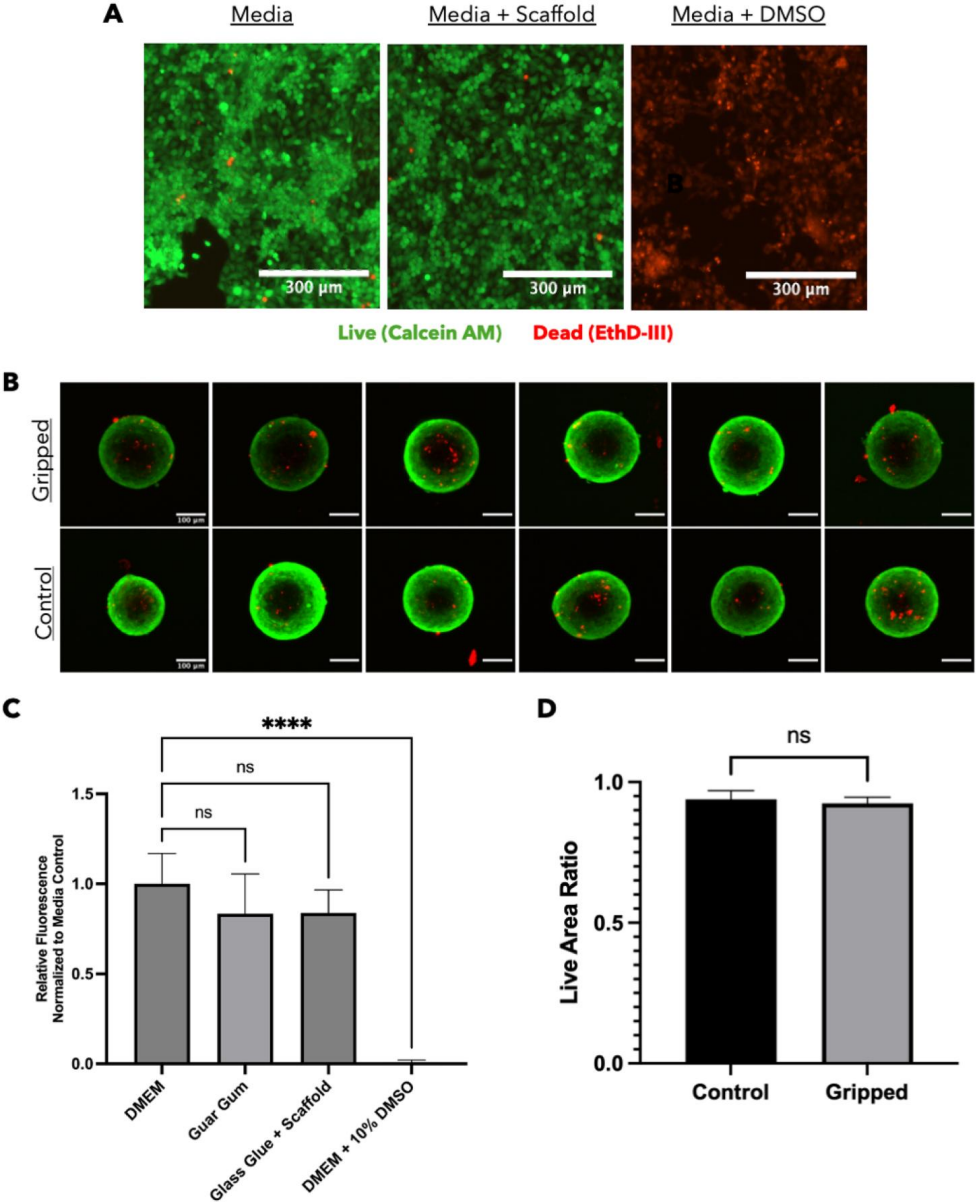
Spheroid seeding conditions and MMG gripping force do not significantly affect cell viability. (a) Live/dead staining of Ca1a cells using Calcein AM (live, green) and ethidium homodimer III (dead, red) for DMEM only, DMEM plus grid scaffold, and DMEM plus 10% dimethyl sulfoxide (DMSO). (b) Confocal images of live/dead stained Ca1a spheroids that were gripped by an MMG. The control group was not gripped by an MMG with n = 6 for each group. (c) PrestoBlue cytotoxicity analysis for DMEM, 1% guar gum media, grid scaffold and glass glue conditioned media, and DMEM plus 10% DMSO. Dunnett's multiple comparisons were used for PrestoBlue analysis with n = 6 for each group. ^****^ = P < 0.001. (d) Live area ratio [live area/(live area + dead area)] determined for both control and gripped spheroids shown in part B. A t-test assuming equal variances was performed to determine differences.

To create the ideal MMG bioassembly environment, we performed all seeding in a micro 3D printed grid scaffold glued to a glass chamber slide. We also supplemented complete Dulbecco's Modified Eagle Medium (DMEM) with guar gum to increase the viscosity to between 30 and 40 cP. The increased viscosity is required because of the low inertia of the microrobots and allows for more controlled robot movements by decreasing the velocity of the microrobots. These conditions were ideal for the placement of cell spheroids using the MMGs, but it was unknown how the cells would respond to the altered growth conditions. Viability analyses were performed to determine if the bioassembly environment or MMGs negatively affected cell survival. Ca1a cells grown in media conditioned with a grid scaffold showed sufficient viability based on live/dead staining [Fig. 5(a)]. PrestoBlue analysis of cells in the grid scaffold and cyanoacrylate glass glue conditioned media or a 1% guar gum media did not show a significant change in viability when compared to the DMEM negative control [Fig. 5(c)]. All experimental conditions and the negative control were significantly different than the dimethyl sulfoxide (DMSO) positive control. Together, these results indicate the bioassembly environment with grid scaffolds, cyanoacrylate, and guar gum will not affect cell survival.

To validate that the MMGs would not damage the cell spheroids, we performed live/dead staining on spheroids gripped by an MMG at an estimated force of 500 *μ*N. This force (500 *μ*N) is much larger than the estimated force from our FEA model, but we wanted to ensure our robots would not damage the spheroids even in the worst gripping conditions. The live/dead images of gripped spheroids showed no obvious mechanical displacement and no increase in dead cells compared to the non-gripped control group [Figs. 5(c) and 5(d)]. Based on these data, we can conclude the MMGs did not cause unwanted damage to the cell spheroids and are safe to use for bioassembly placement. Our method improves on previous spheroid bioassembly techniques because it does not mechanically disrupt the cells or require external hydrogel cross-linking for support.

### D. Microrobotic directed bioassembly

#### 1. Placement

**FIG. 6.**
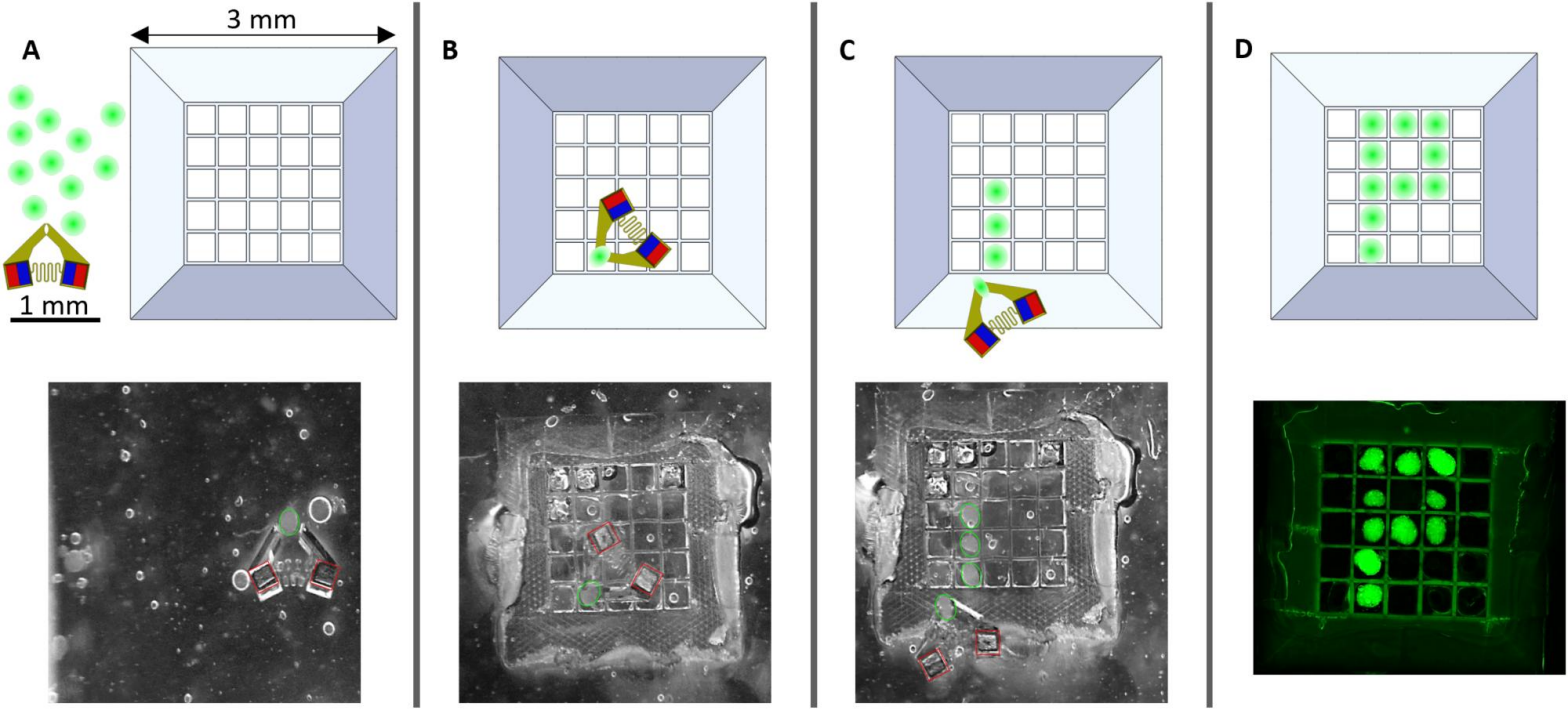
Diagram (top) and microscope images (bottom) of the stages of the bioassembly process. (a) Spheroids and the MMG are placed next to the grid scaffold in media. The grid scaffold is 350 *μ*m tall and features 350 *μ*m cubic holes in a 5 × 5 grid pattern surrounded by a 25° sloped ramp. (b) and (c) Spheroids are individually transported to a desired location in the pattern and placed in the grid scaffold. (d) Completed spheroid pattern. The spheroids then grow and cover the grid scaffold, forming a cohesive tissue.

Fusing spheroids to create tissue constructs is commonly done with metallic skewers or hydrogel constructs, but these methods can cause mechanical damage to the spheroid building blocks or cause undesired external cell signaling. To determine if we could make a tissue construct using magnetic MMGs, we picked and placed 3T3 GFP spheroids into a micro 3D printed grid scaffold. The grid scaffolds were designed with 350 *μ*m square grids with a wall height of 350 *μ*m to fit the cell spheroids and a 25-degree sloped ramp to facilitate MMG access to the top of the grid scaffold. This grid would allow us to make a tissue construct of approximately 3.06 mm^2^. Approximately 10–15 spheroids were manually pipetted into the chamber slide [Fig. 6(a)] with the MMG. Then each spheroid was individually moved into place on the grid scaffold using the MMG [Figs. 6(b) and 6(c)]. For cultures with multiple cell types, spheroids of another cell type were then pipetted into the chamber and placed using the MMG. Spheroid placement times ranged from 5 to 10 min per spheroid. After placing all cells, the guar gum solution was replaced with complete DMEM for continued culture [Fig. 6(d)]. We demonstrate that we can place the spheroids in a designated pattern, allowing for precise spatial control when creating tissue constructs.

#### 2. Cell spreading

**FIG. 7.**
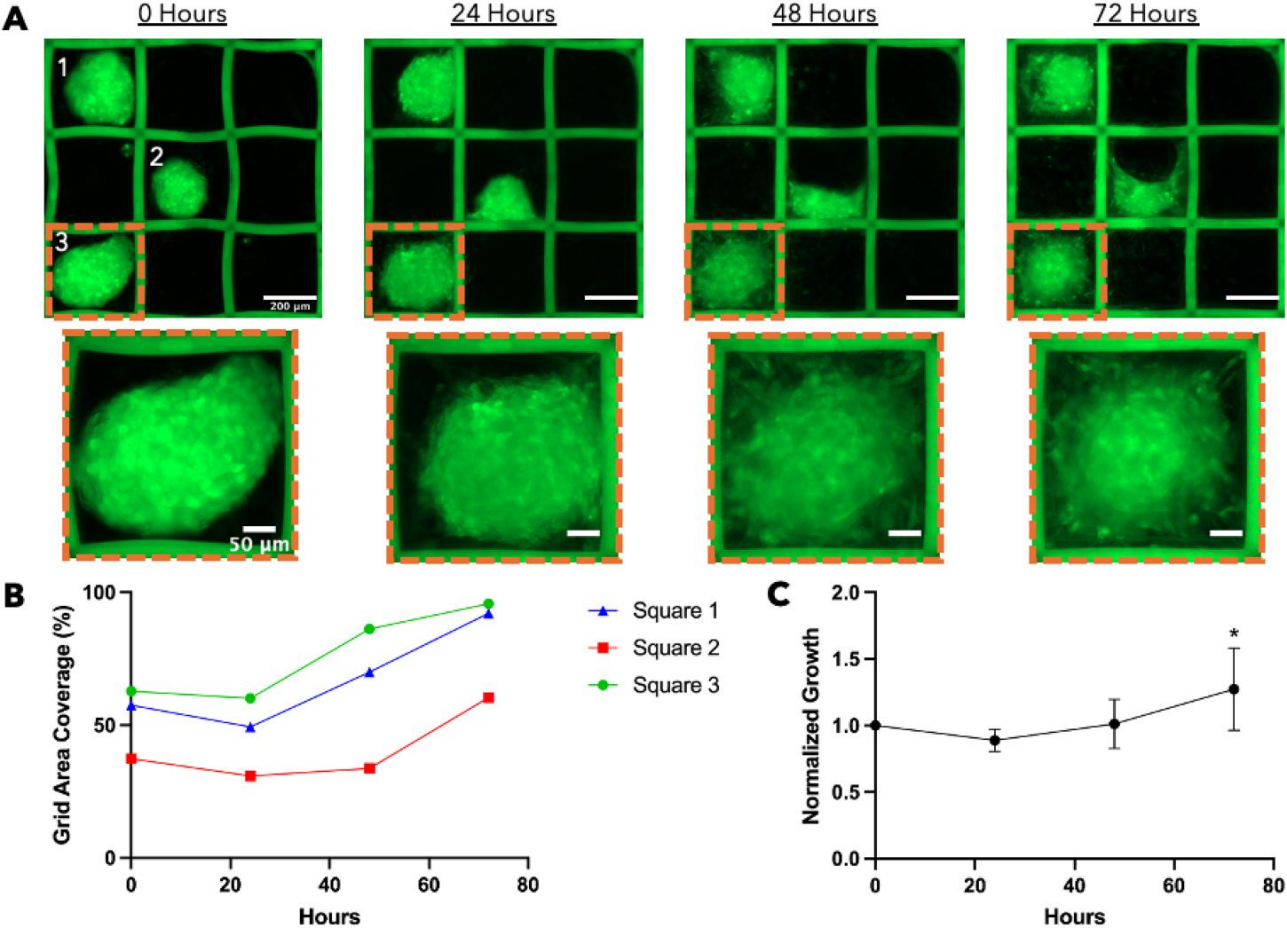
Spheroids placed by MMG will occupy the entire grid space. (a) 3T3 GFP (green) spheroids were placed with an MMG in three separate grids. The 3 × 3 grid was imaged at seeding and every 24 h after seeding. The cell spheroids spread and filled the entire area of a square grid space over time. Scale bars = 200 μm for 3 × 3 grid images and 50 μm for single grid space images. (b) The grid area occupied by the three spheroids in A increased by 72 h. (c) The normalized growth of 3T3 cell spheroids in a grid scaffold over time. N = 8 and * = P = 0.018. All image analyses were performed in ImageJ Fiji.

The entire grid scaffold was imaged at 0, 24, 48, and 72 h to track cell spreading within each grid space [Fig. 7(a)]. Tracking cell spreading with the GFP reporter showed the entire grid space was filled in by the larger spheroids by 72 h [Fig. 7(b)]. Some spheroids, such as spheroid 2 [Fig. 7(a)], did not fill in the grid space by 72 h, but significant spreading did occur. We anticipate this spheroid would have filled the entire grid area if given more time. To determine if there was a trend in cell spreading and growth over time, we accounted for differences in original spheroid size by normalizing each spheroid area to the grid space occupied at 0 h. We found all spheroids followed a similar pattern, where the occupied area decreases at 24 h, then steadily increases over time [Fig. 7(c)]. The initial decrease could be explained by the spheroid attaching to the grid wall and growing vertically rather than outward. We did find a significant difference in normalized spheroid growth between the 0 and 72 h time points, indicating the 3T3 GFP spheroids did spread and occupy more of the grid space. Overall, we showed the MMGs can effectively pick and place cell spheroids into a predefined grid without inhibiting their growth.

#### 3. Co-culture

**FIG. 8.**
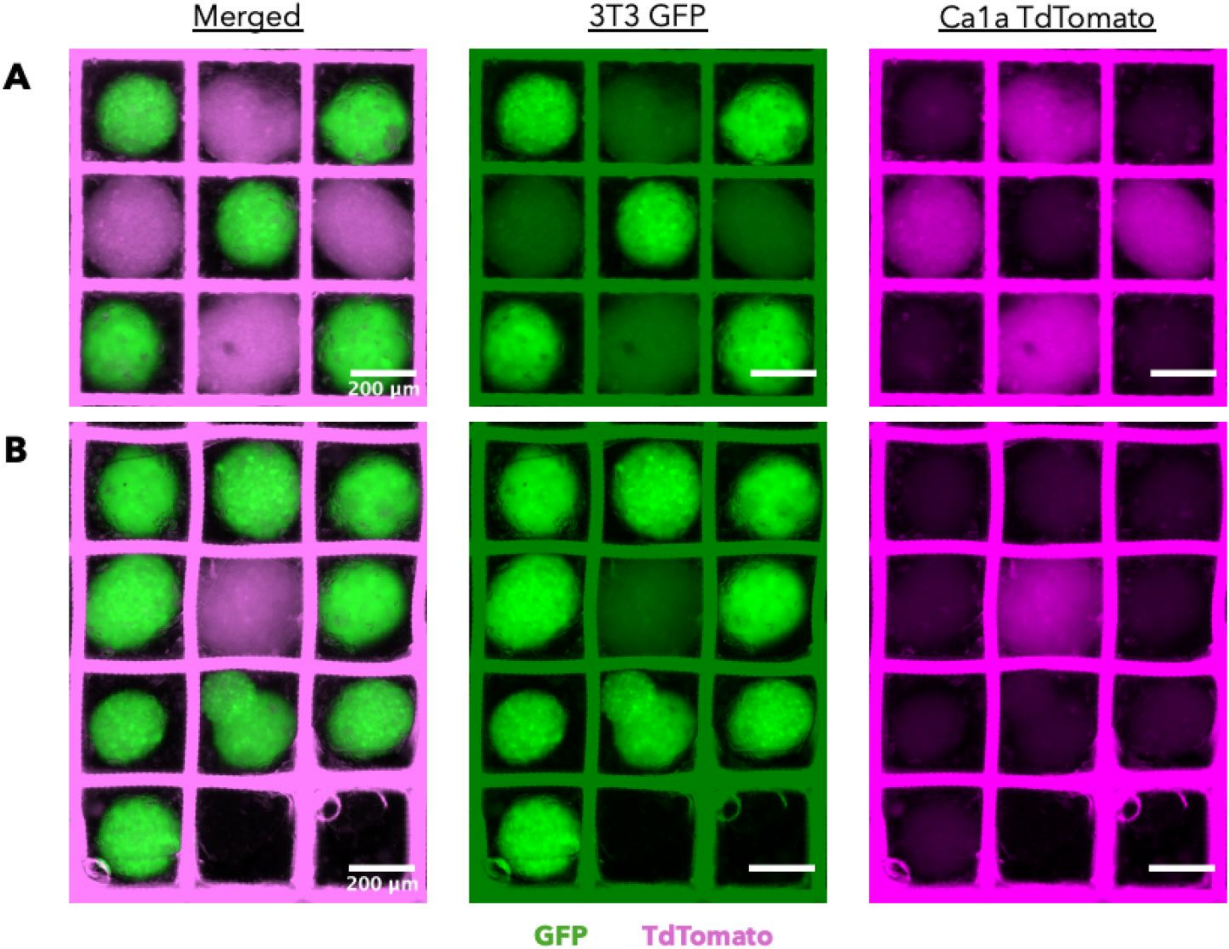
MMGs can control the spatial organization of a spheroid co-culture. 3T3 GFP (green) and Ca1a TdTomato (magenta) spheroids were placed by an MMG in (a) a checkerboard pattern and (b) a “P”-shape pattern in a grid. Images were acquired using a Biotek Cytation 5. Scale bars = 200 μm for all images.

Creating heterogeneous *in vitro* tissue models would be more representative of cell–cell and cell–ECM (extracellular matrix) interactions, increasing their physiological relevance and utility for studying disease progression. Unfortunately, creating these heterogeneous constructs has been a challenge in the field of tissue engineering. To accommodate this need, we used the MMG to pick and place 3T3 GFP and Ca1a-dTom spheroids into a grid scaffold. This method allowed us to precisely control the spatial patterning of the tissue construct, where we created a checkerboard [Fig. 8(a)] and “P”-shaped patterns [Fig. 8(b)]. We demonstrate our method can seed breast cancer cells and fibroblasts together, allowing us to mimic different features of the tumor microenvironment. Although this is just one application, we believe our method could be extended to many cell types to model other disease states or create engineered tissues. In addition, our MMGs could place multiple different cell types into the same grid scaffold, further increasing its relevance as a model to study disease progression or drug response. Our method also has the potential to be used for organoid delivery, expanding its use for regenerative medicine applications.

## III. CONCLUSION

This work presents a novel approach to pick-and-place bioassembly using force-sensing magnetically actuated microgrippers (MMGs) fabricated by two-photon 3D printing. These MMGs offer precise control over spheroid manipulation, enabling the creation of spatially defined multicellular structures without compromising cell viability. We demonstrated that our MMGs, with varying hinge widths resulting in a range of maximum controllable gripping forces up to approximately 136–323 *μ*N, are compatible with cell culture environments and do not negatively impact cell survival. This addresses a key limitation of existing bioassembly methods, such as metallic skewers and hydrogel encapsulation, which can cause mechanical damage or introduce undesired signaling cues. Furthermore, we showcased the versatility of our MMGs by creating both checkerboard and “P” shaped patterns with different cell types, highlighting their potential for constructing complex, heterogeneous tissue models. While this study focused on two cell types, the ability to gently manipulate individual spheroids suggests the potential to incorporate a greater variety of cells in more intricate arrangements. In addition, this work demonstrated we are able to make 2D constructs mimicking heterogeneous cell sheets, but future work plans to expand this to larger 3D constructs by stacking scaffolds to create thicker tissue mimics. One limitation of the current system is the time required for manual manipulation of individual spheroids. Future work will focus on automating the pick-and-place process through integration with the current computer vision and machine learning algorithms to increase throughput and precision. This technology holds significant promise for advancing the field of tissue engineering by enabling the fabrication of *in vitro* models that more accurately mimic the complexity of native tissues. These models have potential in the study of disease progression and drug response as well as the development of regenerative medicine therapies. The ability to precisely manipulate and assemble spheroids using our MMG technology represents a significant step toward building functional tissues and organs in the lab.

## IV. METHODS

### A. MMG fabrication and assembly

**FIG. 9.**
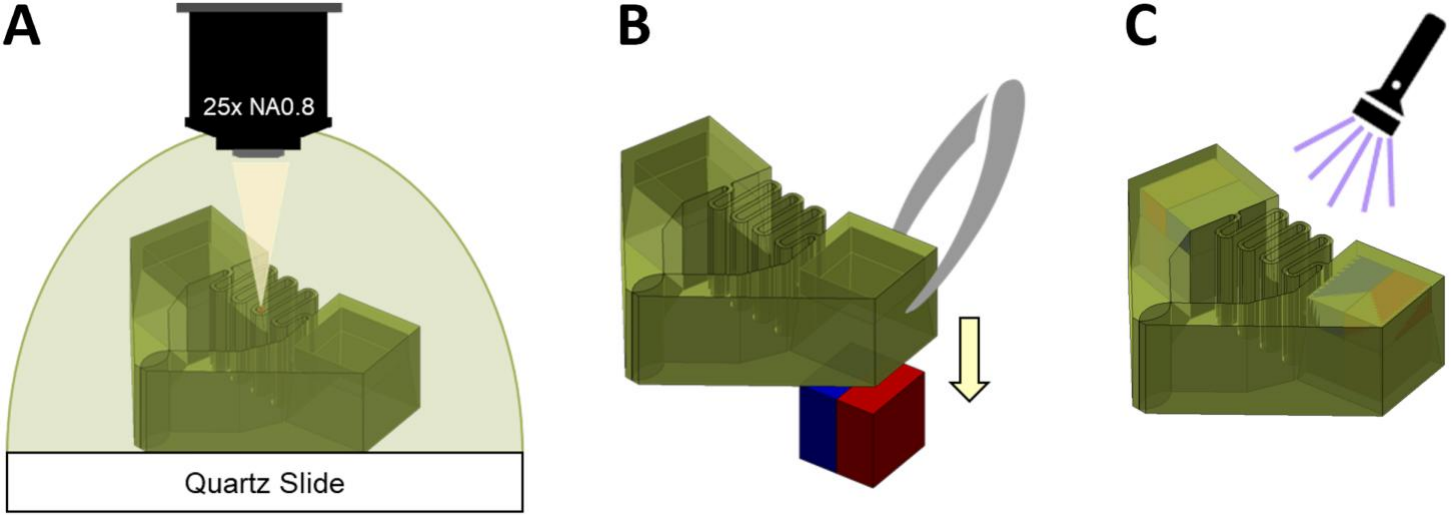
MMG fabrication process. (a) The MMG body is printed using two-photon 3D printing. (b) The MMG is assembled by placing carefully aligned micromagnets into the cavities. (c) The assembled MMG is rinsed in IPA and cured under UV light.

The fabrication of the microgripper bodies was carried out using a two-photon 3D printing technique (Fig. 9). This method allowed for the creation of complex microstructures with high precision. The printing was done using a Nanoscribe Photonic Professional GT2 (PPGT2) printer with a 25× microscope objective and an IP-S resin as the base material. The 3D MF solid slicing recipe was used. The microgripper bodies were printed directly onto indium tin oxide-coated glass substrates and developed in SU-8 developer for 20 min and rinsed in isopropyl alcohol (IPA) for 5 min. The assembly process involved the careful placement of 250 *μ*m cube micromagnets (SM Magnetics) into the cavities of the printed microgripper bodies. The magnets were aligned using a bar magnet to ensure proper orientation and polarity. Fine-tipped, anti-magnetic tweezers attached to a micromanipulation system (MP-225 from Sutter Instruments) were used to manipulate and insert the magnets into the designated cavities. Once the magnets were in place, the assembled microgrippers were rinsed in IPA to remove any excess resin and fully cured under ultraviolet (UV) light for 20 min to solidify the structure and ensure that the magnets were securely held within the cavities.

To enable real-time, vision-based force feedback, the gripper arms are built with high-contrast colored fiducials, which are created by filling cylindrical through-hole cavities with a pigmented UV-curable resin (FormLabs Clear). These fiducials enable a computer vision system to accurately and reliably track the gripper's opening displacement in real-time. More details on the vision feedback system and gripping force measurement are given in our previous work.^57^

Grid scaffolds were also 3D printed with the Nanoscribe PPGT2. The part was sliced using the 3D LF shell parameters and printed with IP-Q resin and a 10× microscope objective on a silicon substrate. Before printing substrates were spin-coated with a 5% dextran release layer at 2000 rpm. The print was developed and cured under UV light following the same parameters as the MMG. The substrate was then submerged in DI water for 5 min to dissolve the dextran film and release the grid scaffolds.

### B. Magnet characterization

The micromagnets were characterized individually by using vibrating-sample magnetometry (PPMS DynaCool from Quantum Design) to measure magnetic moments ( 8.9±2.4 *μ*Am^2^). Magnetic remanence was then calculated for use in FEA analysis ( $B_{r}=714\pm 196$ mT). Variations in the physical dimensions of individual magnets were not measured and may partially explain the large variance in strength between magnets and the relative weakness (50%) compared to the bulk properties of N50 grade NdFeB magnets ( $B_{r}=1.4$ mT).

### C. Gripper calibration

To calibrate the gripper, a range of magnetic fields (0–15 mT) is systematically applied, and the corresponding gripper opening is measured and averaged at each increment. A linear regression is then performed on the collected data to determine the opening coefficient $C_{om}$, which defines the linear relationship between the applied magnetic field and the gripper's displacement.

### D. Finite element analysis

Numerical modeling of the magnetic and mechanical response of the MMGs was done using COMSOL multiphysics. Material properties used were retrieved from the manufacturer for the IP-S resin and from the characterization described above for the magnets.

### E. Cell culture and spheroid growth

Human MCF10CA1a cells stably expressing TdTomato (Ca1a-TdTom) fluorescent protein and NIH 3T3 cells stably expressing GFP (3T3 GFP) were cultured in a humidified incubator at 37 °C with 5% CO_2_ in T-75 culture flasks at a starting cell concentration of 1 × 10^6^ cells. Cultures were maintained with high glucose Dulbecco's Modified Eagle Medium (DMEM), supplemented with 10% fetal bovine serum and 1% penicillin–streptomycin. All cultures were passaged once 80% confluency was reached. TdTomato and GFP cells were used to track cellular growth fluorescently without fixing or staining interference.

The spheroid seeding protocol was adapted from.^6^ Briefly, 2000 Ca1a-TdTom cells in 200 *μ*l of completed high glucose DMEM were seeded into 96 well ultra-low attachment round bottom plates (Corning Spheroid Plate, 4520). 3T3 GFP cells (4000) were seeded in 200 *μ*l of media in the same ultra-low attachment round bottom plates. The 3T3 plates were centrifuged at 1500 rpm for 5 min to encourage spheroid formation. Spheroids ranging from 200 to 300 *μ*m formed after 24 h in culture. All spheroids were seeded in chamber slides for placement by the MMG. Placement occurred between 24 and 48 h after initial seeding.

### F. Environment for robot control and spheroid placement

Grid scaffolds were glued to the bottom of glass chamber slides using glass glue (Loctite, 233841). Viscous media must be used during spheroid placement to better control the MMG motion. DMEM was supplemented with 1% guar gum to increase the viscosity, then 1 ml was added to the well of the chamber slide. Spheroids and the MMG were distributed in the chamber slide well for placement. The MMG was controlled using an 8-coil electromagnetic field generator (MFG-100 from MagnebotiX). Magnetic gradients up to 1500 mT/m were used for translation. Magnetic fields between 1 and 15 mT were used to actuate the gripper opening. The MMG was manually controlled using a 3D mouse (SpaceMouse from 3Dconnexion) and imaged using a microscope camera. The chamber slide was continuously adjusted by hand to keep the MMG in the MFG-100 10 mm diameter work area. Following spheroid placement (approximately 1 h), the 1% guar gum media was replaced with fresh completed DMEM, and the chamber slide was incubated at 37 °C with 5% CO_2_.

### G. Cytotoxicity and viability assays

Cell viability of cells with cured IP-Q (the grid scaffold material) was analyzed using a viability assay kit (Biotium, 30002). The cured IP-Q was incubated in completed DMEM for 24 h to create grid scaffold conditioned media. Ca1a cells were plated at a concentration of 10 000 cells/well in a tissue culture treated 96 well plate and allowed to grow in DMEM for 24 h. The cells were then switched to new completed DMEM (negative control), conditioned media from the grid scaffold, or DMEM with 10% dimethyl sulfoxide (DMSO; positive control). The cells were allowed to incubate in the experimental media for 24 h before live/dead staining (n = 3). For live/dead imaging, 2 *μ*M Calcein AM and 4 *μ*M ethidium homodimer III (EthD-III) was added in serum-free, phenol red-free media to the well. Live/dead images were collected using a Biotek cytation 5. The live and dead area of each spheroid was measured using the threshold feature in ImageJ based on previously published methods to evaluate spheroid viability.^58,59^

Cytotoxicity of the 1% guar gum solution, grid scaffolds, and glass glue was analyzed using PrestoBlue (ThermoFisher, A13261). The grid scaffolds and glass glue were incubated in completed DMEM for 24 h to create grid scaffold and glass glue conditioned media. 3T3 dark cells were plated at a concentration of 10 000 cells/well in a tissue culture treated 96 well plate and allowed to grow in DMEM for 24 h. The cells were then switched to new completed DMEM (negative control), conditioned media from the grid scaffold/glass glue, 1% guar gum, or DMEM with 10% DMSO (positive control). The cells were allowed to incubate in the experimental media for 24 h before PrestoBlue analysis (n = 6). PrestoBlue was diluted 1:10 in serum-free, phenol red-free high modified DMEM, and added to the cells for 30 min before reading the fluorescence using a Biotek Cytation 5.

To determine if manipulation affects cell viability, Ca1a dark spheroids were stained using Calcein AM and EthD-III to spatially visualize live and dead cells. Spheroids were placed in a chamber slide with serum-free DMEM for robot manipulation, and then 2 *μ*M Calcein AM and 4 *μ*M EthD-III were added directly to the chamber slide well. The dye was allowed to incubate at room temperature for 45 min before imaging on a Ziess 880 inverted confocal microscope at a magnification of 10x.

### H. Statistical analysis

One-way ANOVA with Dunnett's multiple comparisons was used to compare the results from the PrestoBlue study (n = 6 for each group). All experimental groups were compared to the negative control (DMEM) at a confidence level of 95%. Tukey's multiple comparisons was used to compare the normalized growth of 3T3 spheroids at 0 and 72 h (N = 8). All analyses were performed using GraphPad Prism.

## SUPPLEMENTARY MATERIAL

See the supplementary material for a video showing representative experimental validation tests. Specifically, the video illustrates examples of MMG spheroid placement and PDMS sphere micromanipulation.

## Data Availability

The data that support the findings of this study are available from the corresponding author upon reasonable request.
